# Magnetic Resonance Imaging Tissue Signatures Associated With White Matter Changes Due to Sporadic Cerebral Small Vessel Disease Indicate That White Matter Hyperintensities Can Regress

**DOI:** 10.1161/JAHA.123.032259

**Published:** 2024-01-31

**Authors:** Angela C. C. Jochems, Susana Muñoz Maniega, Una Clancy, Carmen Arteaga, Daniela Jaime Garcia, Francesca M. Chappell, Will Hewins, Rachel Locherty, Ellen V. Backhouse, Gayle Barclay, Charlotte Jardine, Donna McIntyre, Iona Gerrish, Agniete Kampaite, Eleni Sakka, Maria Valdés Hernández, Stewart Wiseman, Mark E. Bastin, Michael S. Stringer, Michael J. Thrippleton, Fergus N. Doubal, Joanna M. Wardlaw

**Affiliations:** ^1^ Centre for Clinical Brain Sciences University of Edinburgh Edinburgh United Kingdom; ^2^ UK Dementia Research Institute at the University of Edinburgh Edinburgh United Kingdom; ^3^ Edinburgh Imaging Facility, Royal Infirmary of Edinburgh Edinburgh United Kingdom

**Keywords:** cerebral small vessel disease, cerebrovascular disease, imaging, MRI, white matter hyperintensity, Cerebrovascular Disease/Stroke, Ischemic Stroke, Magnetic Resonance Imaging (MRI)

## Abstract

**Background:**

White matter hyperintensities (WMHs) might regress and progress contemporaneously, but we know little about underlying mechanisms. We examined WMH change and underlying quantitative magnetic resonance imaging tissue measures over 1 year in patients with minor ischemic stroke with sporadic cerebral small vessel disease.

**Methods and Results:**

We defined areas of stable normal‐appearing white matter, stable WMHs, progressing and regressing WMHs based on baseline and 1‐year brain magnetic resonance imaging. In these areas we assessed tissue characteristics with quantitative T1, fractional anisotropy (FA), mean diffusivity (MD), and neurite orientation dispersion and density imaging (baseline only). We compared tissue signatures cross‐sectionally between areas, and longitudinally within each area. WMH change masks were available for N=197. Participants' mean age was 65.61 years (SD, 11.10), 59% had a lacunar infarct, and 68% were men. FA and MD were available for N=195, quantitative T1 for N=182, and neurite orientation dispersion and density imaging for N=174. Cross‐sectionally, all 4 tissue classes differed for FA, MD, T1, and Neurite Density Index. Longitudinally, in regressing WMHs, FA increased with little change in MD and T1 (difference estimate, 0.011 [95% CI, 0.006–0.017]; −0.002 [95% CI, −0.008 to 0.003] and −0.003 [95% CI, −0.009 to 0.004]); in progressing and stable WMHs, FA decreased (−0.022 [95% CI, −0.027 to −0.017] and −0.009 [95% CI, −0.011 to −0.006]), whereas MD and T1 increased (progressing WMHs, 0.057 [95% CI, 0.050–0.063], 0.058 [95% CI, 0.050 –0.066]; stable WMHs, 0.054 [95% CI, 0.045–0.063], 0.049 [95% CI, 0.039–0.058]); and in stable normal‐appearing white matter, MD increased (0.004 [95% CI, 0.003–0.005]), whereas FA and T1 slightly decreased and increased (−0.002 [95% CI, −0.004 to −0.000] and 0.005 [95% CI, 0.001–0.009]).

**Conclusions:**

Quantitative magnetic resonance imaging shows that WMHs that regress have less abnormal microstructure at baseline than stable WMHs and follow trajectories indicating tissue improvement compared with stable and progressing WMHs.

**Nonstandard Abbreviations and Acronyms**
DTI: diffusion tensor imaging
FA: fractional anisotropy
FWF: free water fraction
MD: mean diffusivity
NAWM: normal‐appearing white matter
NDI: neurite density imaging
NODDI: neurite orientation dispersion and density imaging
ODI: orientation dispersion imaging
QT1: quantitative T1
SVD: small vessel disease
WMH: white matter hyperintensity

**Clinical Perspective**

**What Is New?**

- The tissue changes in white matter hyperintensities (WMHs) that regress confirm that regression is not a measurement error.
- Regressing WMHs have better microstructural integrity than stable WMHs, before these tissue classes visibly changed on conventional magnetic resonance imaging.
- Regressing WMHs follow different trajectories to progressing and stable WMHs, suggesting potential tissue improvement, whereas other tissue classes are stable or worsen over time.

**What Are the Clinical Implications?**

- This study shows that WMH regression might be a target for new treatments to maintain brain health and prevent clinical decline.
- Prospective validation of the findings and relations to clinical predictors and outcomes is required.

White matter hyperintensities (WMHs) of presumed vascular origin are imaging features of cerebral small vessel disease (SVD).^1^ WMH presence and progression are related to cognitive decline^2^, ^3^, ^4^, ^5^ and an increased risk of stroke, dementia, and death.^6^, ^7^ Although WMH progression and its clinical consequences are widely acknowledged, there is increasing evidence that WMHs can also regress, which might lead to better clinical outcomes.^8^, ^9^ A systematic review of 41 articles (N=12 284) showed that WMH regression occurs in several populations including community‐dwelling people and patients with stroke.^10^ However, the use of total WMH volume change overlooks evidence that individuals can have discrete areas of WMHs that progress and regress contemporaneously. Co‐occurring progression and regression have been found in the general population with sporadic SVD,^11^ patients with Alzheimer disease with SVD,^12^, ^13^ and patients with ischemic stroke.^14^, ^15^ Although WMHs likely represent damaged white matter, it is still unclear why some WMHs remain stable over time and other WMHs regress and seem to become normal‐appearing white matter (NAWM) again.

Pathological examinations of WMHs mention a variable range of features (eg, gliosis, perivascular space dilation, edema, myelin pallor, and inflammation),^16^, ^17^ but the exact cause is not yet fully understood. White matter structure can be noninvasively assessed in vivo with quantitative magnetic resonance imaging (MRI).

Diffusion tensor imaging (DTI) can detect global microstructural white matter changes^18^ before these become visible on conventional structural MRI.^19^ Variables that can be extracted from DTI are fractional anisotropy (FA; representing the degree of directionality of water molecule diffusion) and mean diffusivity (MD; representing the magnitude of water diffusion in all directions). These differ between WMH and NAWM,^20^ with low FA and high MD reflecting impaired microstructural integrity. FA also decreases in NAWM in proximity to WMHs; this phenomenon is called the WMH penumbra.^21^ Neurite orientation dispersion and density imaging (NODDI) is a more complex model, applied to diffusion MRI data, which assumes 3 biophysical compartments in each voxel of the image: intracellular, extracellular, and free water.^22^ Within voxels, NODDI provides more specific descriptions of the tissue, such as the density of neurites (Neurite Density Index [NDI]), orientation of neurites (Orientation Dispersion Index [ODI]), and cerebrospinal fluid (free water fraction [FWF]). NODDI has been widely used as a marker of white matter integrity in aging^23^ and neurological diseases (eg, multiple sclerosis and Alzheimer disease),^24^ stroke,^25^ and psychiatric disorders,^26^ and it provides additional information to DTI.^27^ Quantitative T1 (QT1) relaxation time mapping provides information on brain water content, with longer relaxation times reflecting changes such as edema^28^, ^29^ and WMHs.^30^

Slowing WMH progression has been a target to assess new treatments for many years, and it is not clear whether WMH regression might prevent further clinical decline. More information about the underlying mechanisms and structures of white matter changes would help understand and potentially better target WMH longitudinal change.

In the present study, we examined tissue signatures underlying WMH change within individuals by identifying areas of WMH progression, regression, and stability (ie, stable WMHs and stable NAWM), over 1 year. We used diffusion‐based MRI measures including DTI and NODDI, and QT1, to establish tissue characteristics that might differentiate between the 4 tissue classes (ie, stable NAWM, stable WMHs, progressing WMHs, and regressing WMHs). We hypothesize that, at baseline, areas of WMHs that regress over a year of follow‐up will show characteristics of less structurally damaged tissue than stable WMHs, whereas areas of NAWM that progress into WMHs will be more structurally damaged than stable NAWM. Additionally, we expect that although progressing and stable WMHs will show signatures of accumulated damage over time, regressing WMHs will show slower accrual of damage or potentially signs of recovery.

## Methods

Supporting data of this study are available from the corresponding author upon reasonable request.

### Participants

We recruited patients who presented to the Lothian Stroke Services. Participants were included in a longitudinal observational study (Mild Stroke Study 3; ISRCTN 12113543)^31^ if they were ≥18 years old and had lacunar or minor cortical ischemic stroke; all participants were expected to have a modified Rankin Scale score ≤2 at recruitment. Participants with mild cortical ischemic stroke form the controls to the lacunar stroke participants, because they have similar vascular risk factors and received similar secondary prevention. This accounts for the effect of medication on blood vessel function.^31^ We excluded individuals with severe respiratory, cardiac, or neurological disorders, or when they had MRI contraindications. The stroke diagnosis was made by specialist stroke physicians and neuroradiologists. All study participants gave written informed consent. The Southeast Scotland Regional Ethics Committee (18/SS/0044) approved the study. The corresponding author had full data access.

Participants attended the baseline visit within 3 months of the index stroke and underwent brain MRI. We also recorded medical history and demographic information. All participants were invited back for a visit ≈1 year after their baseline visit for follow‐up MRI.

### Imaging Acquisition

At both the baseline and the 1‐year follow‐up visit, participants underwent brain MRI on the same 3T scanner (MAGNETOM Prisma; Siemens Healthcare, Erlangen, Germany). The full MRI protocol has been published elsewhere.^31^ We acquired images using a 32‐channel head coil (Siemens Healthcare). Briefly, the protocol included the following structural images at both time points: 3‐dimensional (3D) T1‐weighted (1.0 mm^3^ isotropic resolution), 3D T2‐weighted (0.9 mm^3^ isotropic resolution), 3D fluid‐attenuated inversion recovery‐weighted (1.0 mm^3^ isotropic resolution), and 3D proton density imaging (1.2 mm^3^ isotropic resolution).

The following quantitative MRI data were also acquired: QT1, consisting of two 3D inversion‐recovery prepared spoiled gradient echo sequences; 1.2 mm^3^ isotropic resolution, inversion time=600/1500 ms), and three 3D spoiled gradient echo (1.2 mm^3^ isotropic resolution, flip angle=2^°^, 5^°^, 12^°^); multishell diffusion imaging (2.0 mm^3^ isotropic resolution); b=0 s/mm^2^ (15 volumes), b=200 s/mm^2^ (3 volumes), b=600 s/mm^2^ (6 volumes), b=1000 s/mm^2^ (64 volumes), b=2000 s/mm^2^ (64 volumes), and 3 b=0 s/mm^2^ with reverse phase coding. The acquisitions were repeated at 1‐year follow‐up, but with a shorter QT1 sequence (1 inversion‐recovery prepared spoiled gradient echo [inversion time=600 ms] and 2 spoiled gradient echo [2°, 12°]), and single‐shell DTI acquisition (2.0 mm^3^ isotropic resolution; 8 volumes at b=0 s/mm^2^ and 64 volumes at b=1000 s/mm^2^). The baseline QT1 and multishell acquisitions contained the follow‐up quantitative and single‐shell acquisitions, respectively, to allow processing of equivalent quantitative maps at both time points for longitudinal analyses.

Due to the long scanning protocol at baseline and to help participant tolerability, the multishell diffusion sequence was not included in the 1‐year visit protocol, and therefore no NODDI data were available for the 1‐year visit.

The MRI scanner is monitored with a quality assurance program to check for scanner performance issues and to maintain consistent scanner function and image quality.

### Imaging Processing and Analysis

All image sequences were coregistered to the T2‐weighted image using FMRIB's linear image registration tool (FLIRT)^32^ from the FMRIB software library (FSL) (FSL FLIRT).^33^

Intracranial volumes were automatically generated from the coregistered proton density image (or equivalent contrast spoiled gradient echo with flip angle=2° acquired as part of the QT1 acquisition if proton density acquisition was not available) using the brain extraction tool (BET).^34^ Intracranial volumes were checked and manually edited if necessary. NAWM was generated automatically after combining the outputs from FSLFAST (FSL automated segmentation tool) and Freesurfer (https://surfer.nmr.mgh.harvard.edu/); both run using the manually corrected Intracranial volume. WMHs were defined according to the standards for reporting vascular changes on neuroimaging (STRIVE) criteria.^1^ Masks were created by hierarchically thresholding T2‐registered fluid‐attenuated inversion recovery‐weighted images and removing false positives in the vicinities of the choroid plexus, aqueduct, and third and fourth ventricles using Freesurfer. Hyperintense voxels on fluid‐attenuated inversion recovery‐weighted were identified by thresholding intensities to values >1.69 times the standard deviation above the mean intensity of the brain tissue. To exclude hyperintensities unlikely to reflect pathology, a lesion distribution probabilistic template was applied to the threshold images.^35^ Further refinement was achieved by applying Gaussian smoothing, followed by removing voxels with an intensity *Z* score <0.95. The WMH binary masks were inspected and manually corrected for artifact‐related false positives that might have been missed by the automatic pipeline to generate WMH binary masks. These procedures are validated in older people with SVD and mild stroke.^36^, ^37^ Old and acute stroke lesions were manually drawn on the fluid‐attenuated inversion recovery‐weighted sequence by an experienced rater, guided by other MRI sequences including diffusion‐weighted imaging. The rater discussed the stroke lesions for all participants with a neuroradiologist and adjusted the masks if needed. We identified stroke lesions at both visits and excluded those from the WMH volumes and masks to avoid erroneous measures of WMH volume.

### Quantitative T1 Mapping

To account for motion between the scans, all QT1 volumes were coregistered to the first volume using rigid body registration (FSL FLIRT^32^). T1 maps were reconstructed using the driven equilibrium single pulse observation of T1 with high‐speed incorporation of radio frequency field inhomogeneities (DESPOT1‐HIFI) method,^38^ using in‐house code (https://github.com/mjt320/HIFI). This process is described in full elsewhere.^39^

### Diffusion Imaging

We processed diffusion data using TractoR version 3.3.5 dpreproc pipeline.^40^ The digital imaging and communications in medicine (DICOM) data were converted to neuroimaging informatic technology initiative (NIfTI‐1) format using divest,^41^ then corrected for susceptibility and eddy current‐induced distortions using topup and eddy from FSL version 6.0.1.^42^, ^43^, ^44^ FSL's BET was used to mask the brain.^45^

To obtain equivalent DTI both at baseline and the follow‐up visit, we only used the baseline diffusion‐weighted volumes equivalent to the 1‐year single‐shell acquisition (b=0, 1000 s/mm^2^). In each brain voxel, a self‐diffusion tensor model was fitted with TractoR's tensorfit, using an iterative weighted least‐squares approach.^46^ Parametric maps of FA and MD were derived from its eigenvalues.

We fitted NODDI using the full baseline multishell acquisition to calculate NDI, ODI, and FWF with the NODDI toolbox (http://mig.cs.ucl.ac.uk/index.php?n=Tutorial.NODDImatlab).^22^

### White Matter Change Masks

**Figure 1:**
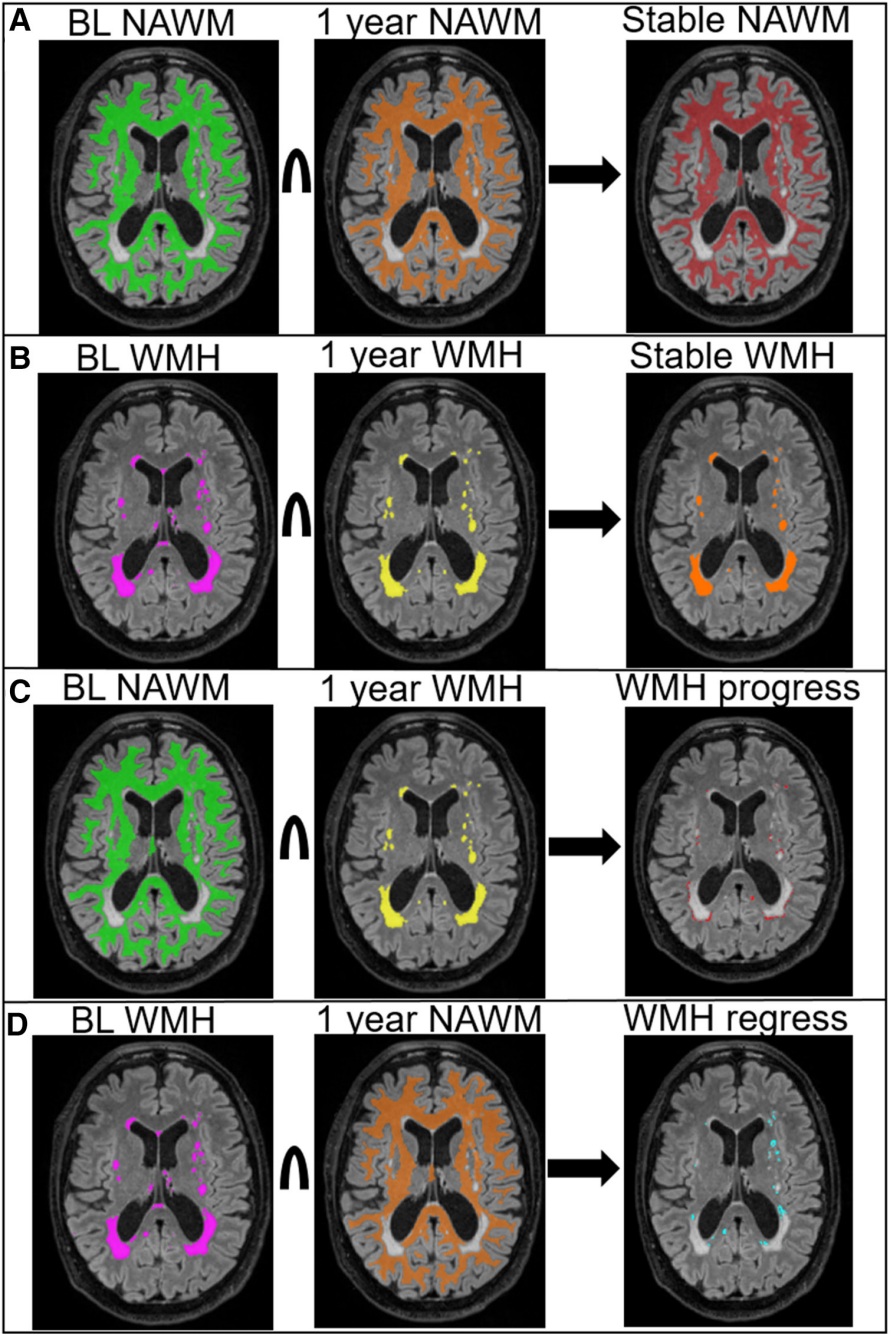
Overview of white matter masks. Areas of stable NAWM (**A**), stable WMHs (**B**), progressing WMHs (**C**), and regressing WMHs (**D**) over 1 year. ∩ indicates intersection; BL, baseline; NAWM, normal‐appearing white matter; and WMH, white matter hyperintensity.

We created masks of white matter changes by using combinations of structural binary NAWM and WMH masks as defined in Figure 1. This resulted in 4 tissue classes: stable NAWM, stable WMHs, progressing WMHs, and regressing WMHs. Areas of stable NAWM (Figure 1A) and stable WMHs (Figure 1B) were classed as the same tissue at baseline and 1 year. We classed progressing WMHs (Figure 1C) as the tissue that was NAWM at baseline but became WMH at 1 year. Regressing WMH masks (Figure 1D) were selected as areas of WMHs at baseline that became NAWM at 1 year.

The masks were registered to the quantitative maps using linear registration between T1‐weighted and the QT1, and nonlinear registration between T2‐weighted and the averaged volumes, with b=0 s/mm^2^ for diffusion, with nearest neighbor interpolation.^30^ Because the masks were derived from structural MRI scans acquired at higher resolution (0.9375×0.9375×0.9 mm^3^) than the diffusion images (2 mm isotropic), the masks might contain clusters of voxels <1 voxel in diffusion space. To reduce partial volume effects, before registration into the diffusion space, we excluded clusters with <5 voxels (structural space), to measure only in clusters at least larger than half a voxel in diffusion space. WMH volume within the masks was calculated in structural space; therefore, no threshold was applied.

### Statistical Analysis

We performed all analyses with R version 4.2.2.^47^ with packages dplyr, car, stats, and emmeans. We created plots with ggplot2. To compare tissue signatures among the 4 tissue classes, we performed 1‐way repeated‐measures ANOVA per quantitative parameter. These analyses were chosen because the quantitative parameters are measures within the 4 different tissue classes within the same individuals. Additional Tukey honestly significant different post hoc analyses were done to identify which tissue classes differed. There were no gross violations of the assumptions. Due to departures from sphericity related to the within‐subjects effect, the Greenhouse‐Geisser correction was applied to the ANOVA results. To examine differences between baseline and 1‐year visit in FA, MD, and T1 per area, we performed paired *t* tests using the Holm method for multiple comparisons correction.

## Results

**Figure 2:**
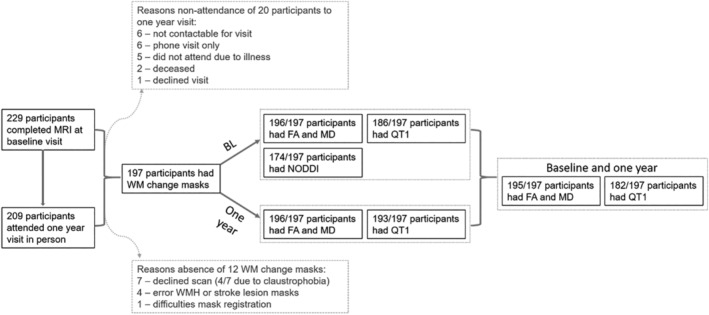
Flow diagram of imaging data collected at baseline and 1‐year visit. BL indicates baseline; FA, fractional anisotropy; MD, mean diffusivity; MRI, magnetic resonance imaging; NODDI, neurite orientation dispersion and density imaging; QT1, quantitative T1; WM, white matter; and WMH, white matter hyperintensity.

At baseline, 229 participants were included in the study, and all participants were invited for a follow‐up visit ≈1 year. See Figure 2 for a flow diagram of attrition and data available. In total, 197 out of 229 underwent MRI at both visits and had useable white matter change masks available. At baseline we gathered FA and MD data for 196 out of 197 participants, NODDI for 174 out of 197, and 186 out of 199 participants had QT1 available.

The mean age at baseline was 65.61 (SD, 11.10) years, 59% had had a lacunar stroke, and 68% of the participants were men (Table 1). After 1 year, 209 out of 229 participants attended the visit (Figure 2). FA and MD measures were available for 198 participants and QT1 for 195. Not all participants who attended both visits had DTI and QT1 data. FA and MD data at both visits were available for 195 and for QT1 for 182 participants. At the 1‐year visit, no NODDI data were available, because there was no multishell diffusion MRI data. All participants had areas of stable NAWM, stable WMHs, and areas of progressing and regressing WMHs (Table 1). WMH progress volumes ranged from 0.05 to 19.25 mL. Regressing WMH volumes ranged from 0.11 to 7.30 mL.

**Table 1:** Overview of Baseline Characteristics and NAWM and WMH Change Volumes Over 1 Year (N=197)

Characteristic	Value
Age, y, mean (SD)	65.61 (11.10)
Male sex, n (%)	133 (67.5)
Lacunar stroke, n (%)	116 (58.9)
Stable NAWM, mL, mean (SD)	360.1 (44.73)
Stable WMH, mL, mean (SD)	12.32 (17.12)
Progressing WMH, mL, mean (SD)	2.98 (3.47)
Regressing WMH, mL, mean (SD)	1.55 (1.53)

NAWM indicates normal‐appearing white matter; and WMH, white matter hyperintensity.

Participants without white matter change masks had smaller baseline WMH volumes (Table S1). There were no differences in age, sex, or infarct subtype.

### Cross‐Sectional Analyses

#### NODDI Baseline Analyses

**Figure 3:**
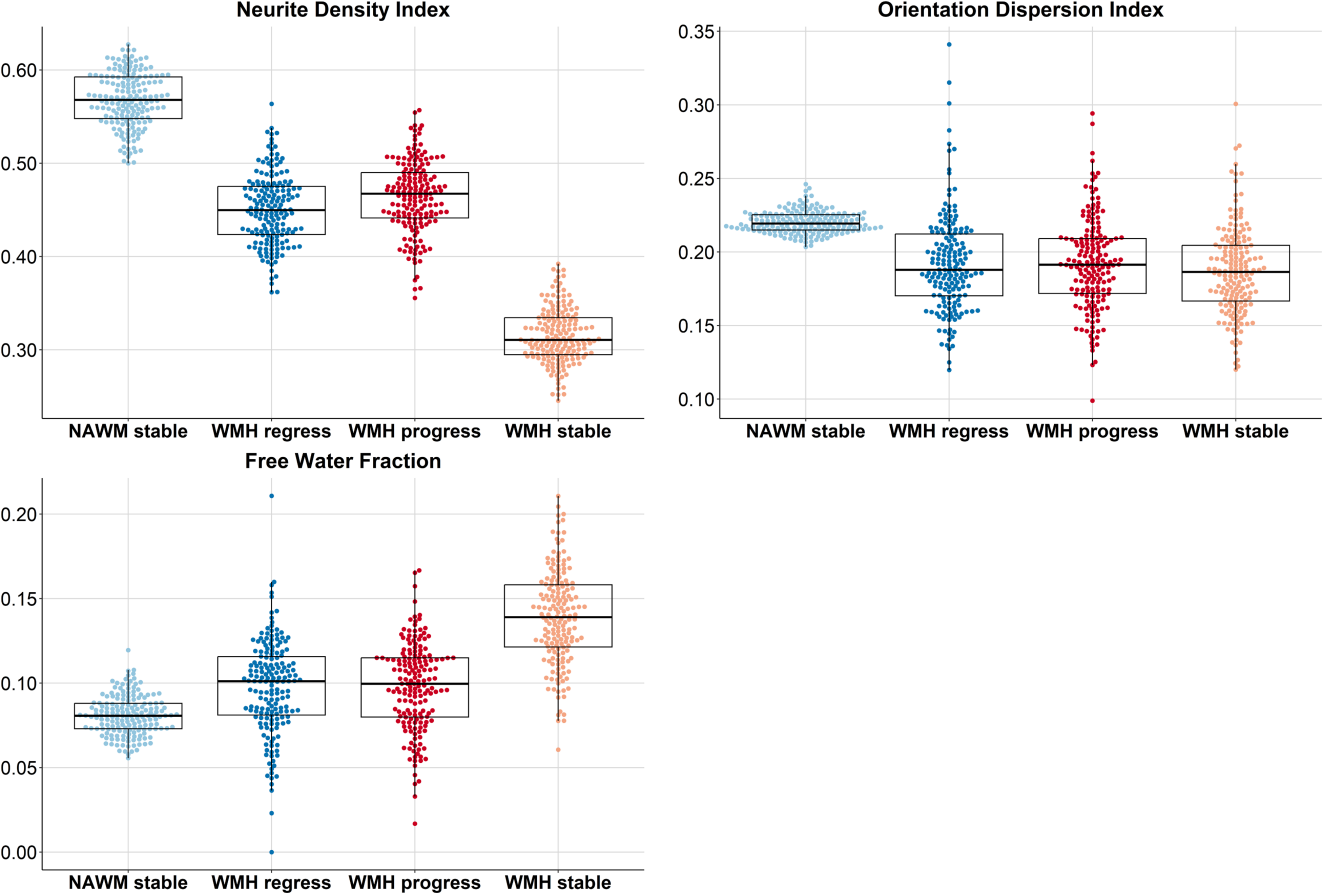
NODDI results at baseline in 4 tissue classes: stable NAWM, stable WMHs, and WMH change. (Top Left) Neurite Density Index. (Top Right) Orientation Density Index. (Bottom Left) Free water fraction. Each boxplot indicates the median and interquartile range for each tissue class. Individual data points are overlaid as a beeswarm. NAWM indicates normal‐appearing white matter; NODDI, neurite orientation dispersion and density imaging; and WMH, white matter hyperintensity.

At baseline, the repeated‐measures ANOVA showed that NDI differed among the 4 tissue classes (*F*[2.8, 488.8]=2684.5; *P*<0.001). Post hoc analyses indicate that all tissue classes differed (Table 2). NDI was highest in NAWM (Figure 3, top left) and lowest for stable WMHs. NDI in WMH progression was slightly higher than in regressing WMHs.

**Table 2:** Overview of Tukey HSD Post Hoc Analyses for Baseline NDI in Areas of White Matter Change

Tissue classes compared	Estimated mean difference	95% CI	Adjusted *P* value
Stable NAWM–progressing WMH	0.103	0.094–0.113	<0.001
Stable NAWM–regressing WMH	0.117	0.108–0.127	<0.001
Stable NAWM–stable WMH	0.253	0.243–0.262	<0.001
Progressing WMH–regressing WMH	0.014	0.004–0.023	0.001
Progressing WMH–stable WMH	0.149	0.140–0.159	<0.001
Regressing WMH–stable WMH	0.136	0.126–0.145	<0.001

HSD indicates honestly significant difference; NAWM, normal‐appearing white matter; NDI, Neurite Density Index; and WMH, white matter hyperintensity.

ODI in NAWM differed from the 3 other areas (*F*[2.6, 443.5]=65.02; *P*<0.001), but the WMH progression, regression, and stable areas did not statistically differ from each other according to post hoc analyses (Table 3 and Figure 3, top right).

**Table 3:** Overview of Tukey HSD Post Hoc Analyses for Baseline ODI in Areas of White Matter Change

Tissue classes compared	Estimated mean difference	95% CI	Adjusted *P* value
Stable NAWM–progressing WMH	0.028	0.021 to 0.036	<0.001
Stable NAWM–regressing WMH	0.028	0.020 to 0.036	<0.001
Stable NAWM–stable WMH	0.033	0.025 to 0.041	<0.001
Progressing WMH–regressing WMH	−0.000	−0.008 to 0.007	>0.999
Progressing WMH–stable WMH	0.005	−0.003 to 0.012	0.394
Regressing WMH–stable WMH	0.005	−0.003 to 0.013	0.351

HSD indicates honestly significant difference; NAWM, normal‐appearing white matter; ODI, Orientation Dispersion Index; and WMH, white matter hyperintensity.

The repeated‐measures ANOVA showed differences of FWF between tissue classes (*F*[2.9, 498.3]=350.32; *P*<0.001). Post hoc analyses showed no statistical differences between regressing and progressing WMHs (Table 4; Figure 3, bottom left). FWF was lowest in NAWM and highest in stable WMHs.

**Table 4:** Overview of Tukey HSD Post Hoc Analyses for Baseline FWF in Areas of White Matter Change

Tissue classes compared	Estimate mean difference	95% CI	Adjusted *P* value
Stable NAWM–progressing WMH	−0.017	−0.024 to −0.010	<0.001
Stable NAWM–regressing WMH	−0.017	−0.023 to −0.010	<0.001
Stable NAWM–stable WMH	−0.058	−0.065 to −0.051	<0.001
Progressing WMH–regressing WMH	0.000	−0.006 to 0.007	0.998
Progressing WMH–stable WMH	−0.041	−0.048 to −0.034	<0.001
Regressing WMH–stable WMH	−0.041	−0.048 to −0.035	<0.001

FWF indicates free water fraction; HSD, honestly significant difference; NAWM, normal‐appearing white matter; and WMH, white matter hyperintensity.

#### FA Baseline and 1‐Year Analyses

**Figure 4:**
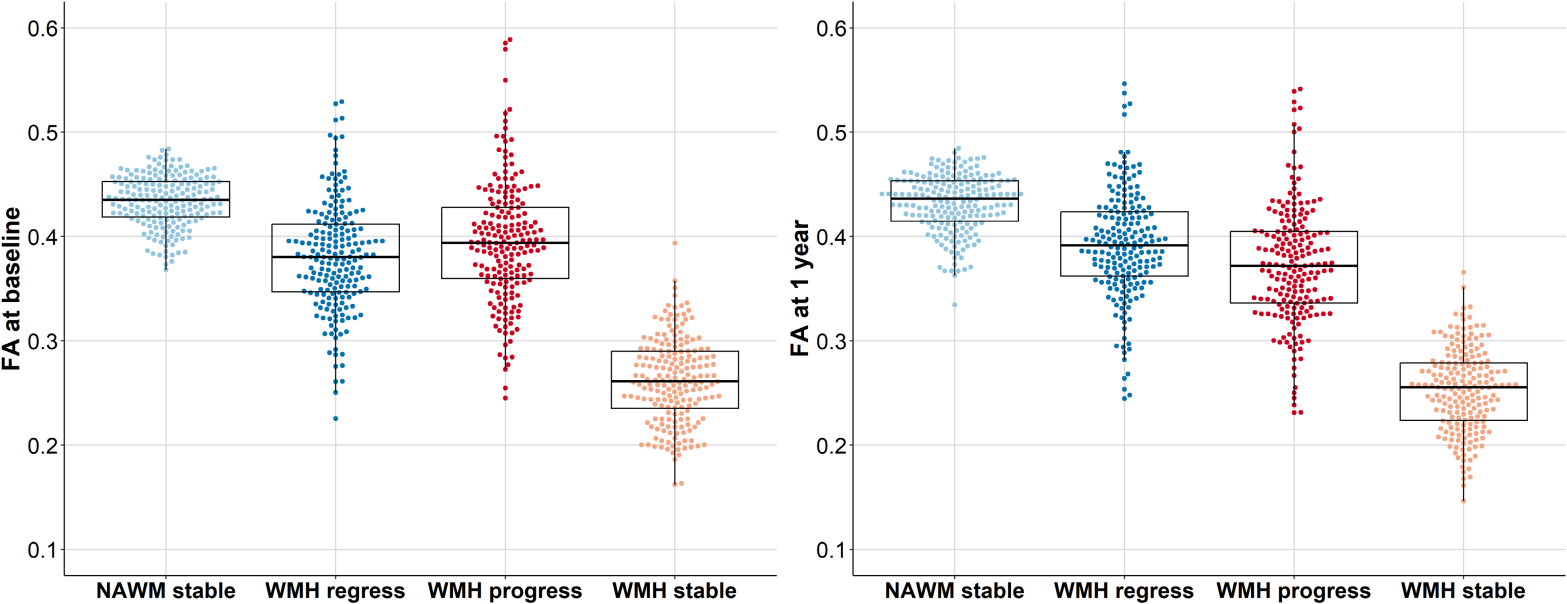
FA baseline (left) and 1‐year values (right) in stable NAWM, stable WMHs, and areas of WMH change. Each boxplot indicates the median and interquartile range for each tissue class. Individual data points are overlaid as a beeswarm. FA indicates fractional anisotropy; NAWM, normal‐appearing white matter; and WMH, white matter hyperintensity.

One‐way repeated‐measures ANOVA at baseline showed differences in FA among all 4 tissue classes (*F*[2.5, 479.0]=573.34; *P*<0.001) (Figure 4; Table 5 for post hoc analyses), with FA being highest in stable NAWM and lowest in stable WMHs (Figure 4, left), whereas FA in areas of progressing WMHs was higher than in regressing WMHs.

**Table 5:** Overview of Tukey HSD Post Hoc Analyses for Baseline and 1‐Year FA in the 4 Tissue Classes

Tissue classes compared	Estimated mean difference	95% CI	Adjusted *P* value
Baseline			
Stable NAWM–progressing WMH	0.039	0.027 to 0.051	<0.001
Stable NAWM–regressing WMH	0.053	0.041 to 0.065	<0.001
Stable NAWM–stable WMH	0.172	0.160 to 0.184	<0.001
Progressing WMH–regressing WMH	0.014	0.002 to 0.026	0.010
Progressing WMH–stable WMH	0.133	0.121 to 0.145	<0.001
Regressing WMH–stable WMH	0.119	0.107 to 0.131	<0.001
1 y			
Stable NAWM–progressing WMH	0.059	0.047 to 0.071	<0.001
Stable NAWM–regressing WMH	0.041	0.028 to 0.052	<0.001
Stable NAWM–stable WMH	0.179	0.167 to 0.191	<0.001
Progressing WMH–regressing WMH	−0.019	−0.031 to −0.007	<0.001
Progressing WMH–stable WMH	0.120	0.108 to 0.132	<0.001
Regressing WMH–stable WMH	0.139	0.127 to 0.151	<0.001

FA indicates fractional anisotropy; HSD, honestly significant difference; NAWM, normal‐appearing white matter; and WMH, white matter hyperintensity.

After 1 year, all tissue classes still differed (*F*[2.6, 511.9]=665.91; *P*<0.001) (Table 5 for post hoc analyses), with FA higher in areas of regressing WMHs compared with progressing WMHs, opposite of baseline values (Figure 4, right).

#### MD Baseline and 1‐Year Analyses

**Figure 5:**
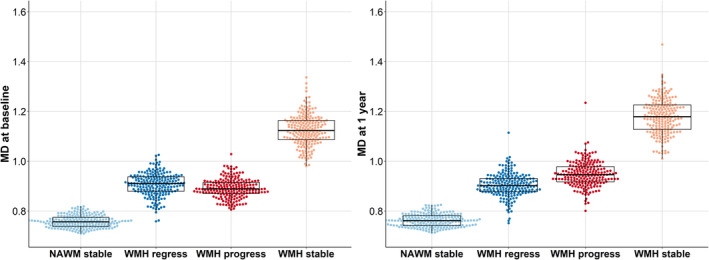
MD (×10^−3^ mm^2^/s) at baseline (left) and at 1 year (right) in stable NAWM, stable WMHs, and WMH change. Each boxplot indicates the median and interquartile range for each tissue class. Individual data points are overlaid as a beeswarm. MD indicates mean diffusivity; NAWM, normal‐appearing white matter; and WMH, white matter hyperintensity.

Similarly, 1‐way repeated‐measures ANOVA at baseline showed differences in MD among the tissue classes (*F*[2.3, 440.1]=3291.3; *P*<0.001) (Figure 5, left). Post hoc analyses confirmed that there were differences between all tissue classes (Table 6). MD was lowest in stable NAWM and highest in stable WMHs, with progressing and regressing WMHs having intermediate values, with progressing WMHs being lower than regressing WMHs.

**Table 6:** Overview of Tukey HSD Post Hoc Analyses for Baseline and 1‐Year MD (×10^−3^ mm^2^/s) in the 4 Tissue Classes

Tissue classes compared	Estimated mean difference	95% CI	Adjusted *P* value
Baseline			
Stable NAWM–progressing WMH	−0.133	−0.145 to −0.121	<0.001
Stable NAWM–regressing WMH	−0.148	−0.160 to −0.136	<0.001
Stable NAWM–stable WMH	−0.367	−0.379 to −0.356	<0.001
Progressing WMH–regressing WMH	−0.015	−0.027 to −0.003	0.006
Progressing WMH–stable WMH	−0.234	−0.246 to −0.223	<0.001
Regressing WMH–stable WMH	−0.219	−0.231 to −0.208	<0.001
1 y			
Stable NAWM–progressing WMH	−0.186	−0.199 to −0.172	<0.001
Stable NAWM–regressing WMH	−0.141	−0.155 to −0.128	<0.001
Stable NAWM–stable WMH	−0.417	−0.430 to −0.404	<0.001
Progressing WMH–regressing WMH	0.044	0.031 to 0.058	<0.001
Progressing WMH–stable WMH	−0.231	−0.245 to −0.218	<0.001
Regressing WMH–stable WMH	−0.276	−0.289 to −0.262	<0.001

HSD indicates honestly significant difference; MD, mean diffusivity; NAWM, normal‐appearing white matter; and WMH, white matter hyperintensity.

At 1 year, ANOVA results showed that MD among all 4 tissue classes still differed (*F*[2.1, 411.9]=3036; *P*<0.001) (Figure 5, right; Table 6 for post hoc analyses). MD was lowest in stable NAWM and highest in stable WMHs at 1 year. MD for progressing WMHs was higher than in regressing WMHs.

#### Quantitative T1 Baseline and 1‐Year Analyses

**Figure 6:**
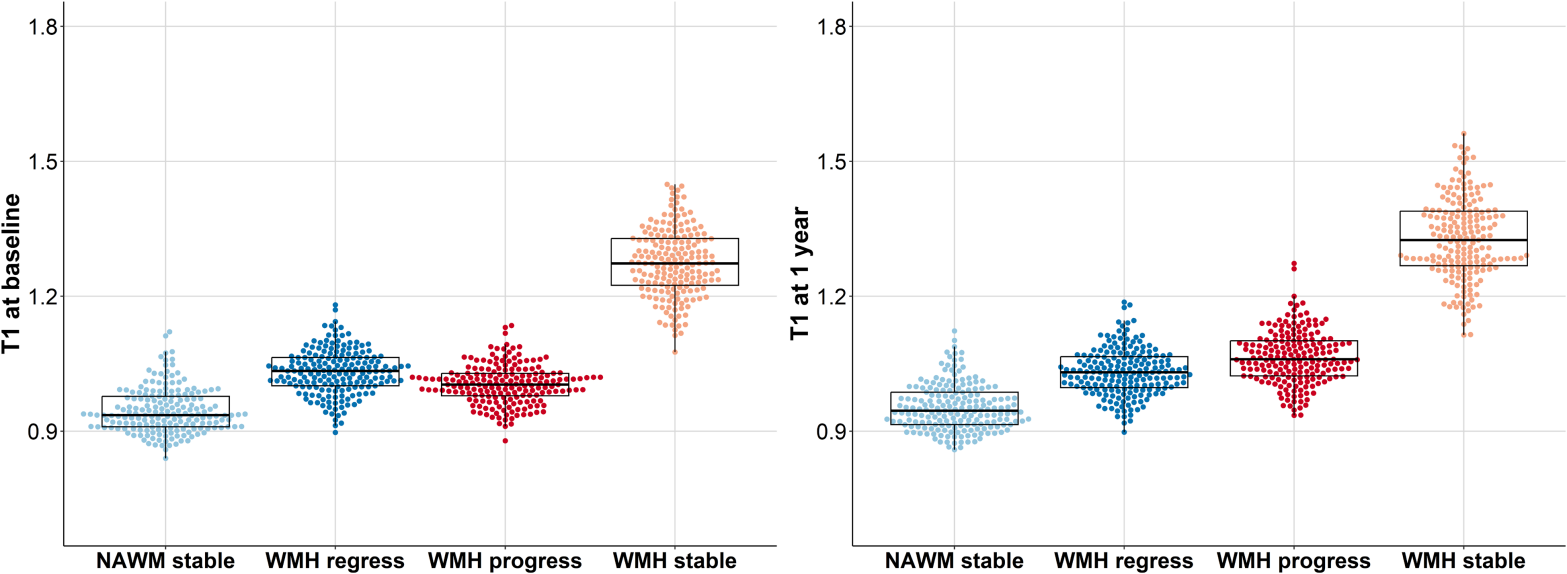
T1 (seconds) at baseline (left) and 1 year (right) in stable NAWM, stable WMHs, and WMH change. Each boxplot indicates the median and interquartile range for each tissue class. Individual data points are overlaid as a beeswarm. NAWM indicates normal‐appearing white matter; and WMH, white matter hyperintensity.

One‐way repeated‐measures ANOVA at baseline showed differences in T1 among the 4 tissue classes (*F*[1.8, 332.6]=2924.5; *P*<0.001) (Figure 6, left). Post hoc analyses showed differences among all 4 classes (Table 7). At baseline, T1 was highest in stable WMHs and lowest in stable NAWM. T1 was lower for progressing WMHs than for regressing WMHs.

**Table 7:** Overview of Tukey HSD Post Hoc Analyses for Baseline and 1‐Year T1 (Seconds) in the 4 Tissue Classes

Tissue classes compared	Estimated mean difference	95% CI	Adjusted *P* value
Baseline			
Stable NAWM–progressing WMH	−0.058	−0.073 to −0.043	<0.001
Stable NAWM–regressing WMH	−0.086	−0.101 to −0.071	<0.001
Stable NAWM–stable WMH	−0.329	−0.344 to −0.314	<0.001
Progressing WMH–regressing WMH	−0.028	−0.043 to −0.013	<0.001
Progressing WMH–stable WMH	−0.271	−0.286 to −0.256	<0.001
Regressing WMH–stable WMH	−0.243	−0.258 to −0.228	<0.001
1 y			
Stable NAWM–progressing WMH	−0.111	−0.128 to −0.094	<0.001
Stable NAWM–regressing WMH	−0.078	−0.095 to −0.061	<0.001
Stable NAWM–stable WMH	−0.374	−0.392 to −0.357	<0.001
Progressing WMH–regressing WMH	0.033	0.016 to 0.050	<0.001
Progressing WMH–stable WMH	−0.263	−0.280 to −0.246	<0.001
Regressing WMH–stable WMH	−0.296	−0.313 to −0.279	<0.001

HSD indicates honestly significant difference; NAWM, normal‐appearing white matter; and WMH, white matter hyperintensity.

At 1 year, T1 still differed between all tissue classes (F[1.9, 368.0]=2800.1; *P*<0.001), confirmed by post hoc analyses (Table 7). Highest T1 was in stable WMHs (Figure 6, right) and lowest in stable NAWM. Progressing WMHs had higher T1 than regressing WMHs. This is different compared with baseline (Figure 6, left), where values in areas of regressing WMHs were higher than in progressing WMHs.

#### Baseline and 1‐Year Comparisons

Paired *t* tests assessing the differences between baseline and 1 year (Table 8) showed that FA decreased and MD and T1 increased in progressing and stable WMHs. For stable NAWM, MD and T1 also increased, whereas FA decreased (estimated mean difference, −0.002[95% CI, −0.004 to −0.000).

**Table 8:** Paired *t* Test Results of Difference Between Baseline and 1 Year for FA, MD, and T1

Tissue class	Estimated mean difference	95% CI	*P* value	Adjusted* *P* value
FA, n=195				
WMH progress	−0.022	−0.027 to −0.017	8.99^−15^	<0.001
WMH regress	0.011	0.006 to 0.017	3.28^−5^	<0.001
WMH stable	−0.009	−0.011 to −0.006	6.59^−11^	<0.001
NAWM stable	−0.002	−0.004 to −0.000	0.017	0.068
MD (×10^−3^ mm^2^/s), n=195				
WMH progress	0.057	0.050 to 0.063	6.07^−41^	<0.001
WMH regress	−0.002	−0.008 to 0.003	0.363	0.726
WMH stable	0.054	0.045 to 0.063	2.05^−25^	<0.001
NAWM stable	0.004	0.003 to 0.005	1.11^−12^	<0.001
T1, s, n=182				
WMH progress	0.058	0.050 to 0.066	1.01^−30^	<0.001
WMH regress	−0.003	−0.009 to 0.004	0.412	0.726
WMH stable	0.049	0.039 to 0.058	6.23^−20^	<0.001
NAWM stable	0.005	0.001 to 0.009	0.020	0.068

FA indicates fractional anisotropy; MD, mean diffusivity; NAWM, normal‐appearing white matter; and WMH, white matter hyperintensity.

* Adjusted for multiple comparisons using the Holm method.

On the other hand, in regressing WMHs, FA increased, whereas MD and T1 showed no change.

## Discussion

In this study, we found that tissue signatures differ between regressing WMHs and progressing WMHs and stable WMHs and NAWM within a population of individuals with minor ischemic stroke at baseline and 1 year later. These signatures can be measured before damage is visible on conventional MRI and suggest that these are different tissue states. They also support the theory that WMHs can regress despite having been regarded by some as a measurement error in the past.^10^ At baseline, WMHs that will progress already have worse microstructural integrity than stable NAWM, whereas regressing WMHs have better microstructural integrity than stable WMHs. This suggests an intermediate stage where tissue could regress or progress away from or toward more permanent damage, offering an opportunity to push tissue toward recovery if interventions can be identified.

WMHs and NAWM tissue signatures are known to differ, reflected by lower FA and higher MD and T1 in WMHs than NAWM.^30^, ^48^ Several studies have also observed the transition between visibly damaged white matter to NAWM as a gradient of these tissue signatures in the WMH penumbra.^21^, ^30^ Although some incident WMHs can emerge as new lesions, the vast majority appear as an extension to existing lesions.^49^ The growing and regressing WMHs are therefore located mostly in the WMH penumbra, and this is reflected by the in‐between values of the quantitative parameters we observe.

Based on the longitudinal changes of FA, MD, and T1, we see that the structure of progressing WMHs, stable WMHs, and even stable NAWM will deteriorate over time. The tissue could become more severely damaged (ie, in progressing WMHs the damage becomes visible on conventional MRI). We also see in general that FA decreases and MD increases with aging.^50^, ^51^, ^52^ However, we observe a different pattern of changes over time in regressing WMHs. In particular, FA suggests that regressing WMHs might structurally improve, despite not becoming fully normal. Although we cannot make strong inferences about the changes occurring at the microstructural level from FA, because its interpretation is limited in areas containing complex white matter fiber geometries or multiple crossing fibers,^53^ we demonstrate that areas of WMH regression follow a different trajectory over time than other white matter areas for FA, MD, and T1.

There is less information from previous work about NODDI applied to SVD lesions. Application of NODDI in multiple sclerosis^54^ suggests lower NDI in WMHs compared with NAWM, and an overall lower NDI in white matter (both WMHs and NAWM) compared with healthy controls. Previous ODI results were inconsistent, and FWF might be higher in WMHs than NAWM.^54^ We see comparable results for NDI and FWF (ie, NDI lowest and FWF highest in WMH, and higher ODI in stable NAWM than the other areas). The intermediate values of NDI and FWF for regressing and progressing WMHs confirm those observed in FA and MD.

Few studies have looked at WMH regression in general.^55^ One study that assessed total WMH volume change found that net WMH volume regression was associated with higher FA.^56^ Another study looked at areas of WMH change in relation to diffusion imaging.^13^ In people with Alzheimer disease (N=5), mild cognitive impairment (N=16), and cognitively intact older people (N=19), there were similar FA and MD results for progressing WMHs and stable WMHs (decrease and increase over 2 years, respectively).^13^ FA also decreased in stable NAWM, but MD also seemed to decrease. Although their MD results for NAWM were not traditionally statistically significant, it is interesting because MD did increase over time.^13^ In that study, they did not find suggestions that regressing WMHs had improved. Their different results could be a result of the small sample size and different population, or due to the different approach used to creating the tissue masks. In our study we made sure that regressing WMH areas were classified as WMHs at baseline and NAWM at 1 year. This both avoided the inclusion of areas of shrinking periventricular WMHs due to enlargement of the lateral ventricles and ensured that the areas of regression appeared normal after 1 year. We also applied voxel cluster thresholding to reduce partial volume effects in the quantitative measurements due to the difference in image resolution. In the study on people with Alzheimer disease, mild cognitive impairment, or no cognitive difficulties, as previously mentioned, regressing WMHs were defined as WMHs at baseline and not in the follow‐up scan,^13^ and although the authors removed areas around the ventricular wall, some global effects of tissue displacement due to atrophy might remain. The fact that our WMH regression masks were specific to normal‐appearing tissue, rather than disappearing damage, could explain the apparent improvement we observed in the quantitative measurements.

Quantitative measures are only estimates of underlying tissue structure. Although it is tempting to discuss results directly related to pathology (eg, demyelination), it remains unknown what MRI markers exactly measure, and it might be best to be cautious (recommended terms to use and avoid^55^). Unfortunately, pathology related to quantitative measures in SVD has been understudied and needs to be further investigated. One histopathological study in patients with Alzheimer disease showed that areas of WMH had more axonal and myelin loss than NAWM. In the same patients, postmortem MRI showed lower FA and higher QT1 values in WMHs than in NAWM. FA correlated with neuropathological findings of axonal loss, and T1 correlated with axonal loss, myelin loss, and microglial activation.^57^

Strengths of this study are the longitudinal design and large sample size at both time points. The application of several measures, within the same NAWM and WMH masks at both visits, to assess microstructural integrity and water content confirm previous findings and provide new insights. NODDI and T1 have not yet been widely applied in relation to areas of changing white matter and WMHs in SVD.

A limitation of this study is that we were not able to compare the NODDI measures at 1 year poststroke, because no multishell diffusion data were acquired at the 1‐year visit. Future work should aim to corroborate the increase of FA we observed in regressing WMHs with measures derived from advanced diffusion models more robust to crossing fibers. Another potential weakness is that the masks, in particular the masks for areas of WMH regression and progression, are small. This makes the measures susceptible to noise and partial volume effects. However, we were able to observe different patterns of change for DTI and T1 values in regressing WMHs compared with other tissues. We have not performed any spatial assessments of WMH progression and regression. We examined progressing and regressing WMHs out of context with surrounding structures (eg, grouped all progressing and regressing WMHs) and did not assess whether they are nearby existing WMHs or isolated in NAWM. This might be relevant, because WMHs might affect nearby white matter and subsequently DTI measures.^21^, ^58^, ^59^ The final limitation is that we examined a stroke population, and these results might not be generalizable to covert SVD.

Our study supports findings that individuals have co‐occurring WMH progression and regression. In general, discrete WMH change is assessed with total WMH (volume) change.^10^ Net WMH volume regression has been found in 4% of sporadic SVD over a 14‐year period, and in between visits during those 14 years, more participants showed WMH volume regression followed by WMH volume progression.^60^ Future studies should assess co‐occurrence of WMH progression and regression over a longer period and examine whether areas of regressing WMHs remain normal appearing or return to WMHs, as well as what risk factors are associated with these changes and what the microstructural integrity of these areas is in nonstroke populations with sporadic SVD. It would be interesting to see how much progression and regression of WMHs occurs in people with total WMH volume increase or decrease. In addition, any studies into clinical and cognitive long‐term outcomes related to WMH regression to establish whether areas of WMH regression and total WMH volume regression have symptomatic benefits should be encouraged.

In this study, we examined FA, MD, and T1 over 1 year and NODDI at baseline in areas of progressing and regressing WMHs and stable WMHs and NAWM in sporadic SVD. The results suggest that WMH regression can occur, and these areas are more microstructurally intact than stable WMH. Over 1 year, the measurements within regressing WMHs follow different trajectories than progressing WMHs and stable WMHs, indicating no deterioration or perhaps some improvement of tissue for regressing WMHs. Although findings need to be replicated and clinical factors in relation to regression still need to be examined, WMH regression is not a measurement error and is a promising potential target for interventions.

## Sources of Funding

This research was funded by the UK Dementia Research Institute, which receives its funding from DRI Ltd. funded by the UK Medical Research Council, Alzheimer's Society, and Alzheimer's Research UK; the Fondation Leducq Network for the Study of Perivascular Spaces in Small Vessel Disease (16 CVD 05); Stroke Association Small Vessel Disease‐Spotlight on Symptoms (SAPG 19n100068); British Heart Foundation Edinburgh Centre for Research Excellence (RE/18/5/34216); the Row Fogo Charitable Trust Centre for Research into Aging and the Brain. A.C.C.J. was funded by the Alzheimer's Society (ref 486 [AS‐CP‐18b‐001]), University of Edinburgh College of Medicine and Veterinary Medicine, and the UK Dementia Research Institute, which receives funding from UK DRI Ltd. as described above. S.M.M. was funded by the Biotechnology and Biological Sciences Research Council, and the Economic and Social Research Council (BB/W008793/1). M.J.T. received funding from the National Health Service Lothian Research and Development Office. U.C. was funded by a Chief Scientist Office Clinical Academic Fellowship (CA/18) and is funded by the Scottish Clinical Excellence Research Development Scheme at the University of Edinburgh. D.J.G. is funded by the Wellcome Trust. C.A. receives funding from Mexican Council of Humanities Science and Technology, Anne Rowling Regenerative Neurology Clinic, and the Row Fogo Charitable Trust Centre into Aging and the Brain. The University 3T MRI Research scanner in the Royal Infirmary of Edinburgh is supported by the Scottish Funding Council through the Scottish Imaging Network, a Platform for Scientific Excellence (SINAPSE) collaboration; the Wellcome Trust (104 916/Z/14/Z), Dunhill Trust (R380R/1114), Edinburgh and Lothians Health Foundation (2012/17), Muir Maxwell Research Fund, Edinburgh Imaging, and the University of Edinburgh. For the purpose of open access, the author has applied a Creative Commons Attribution (CC BY) license to any author‐accepted article version arising.

## Disclosures

None.

## Supporting information

Table S1
