# Supplementary material for: Magnetic Resonance Imaging Tissue Signatures Associated With White Matter Changes Due to Sporadic Cerebral Small Vessel Disease Indicate That White Matter Hyperintensities Can Regress
Source: J Am Heart Assoc. 2024 Jan 31;13(3):e032259. doi: 10.1161/JAHA.123.032259 (PMC11056146; doi:10.1161/JAHA.123.032259)
Supplement: Supplementary file 1 — Table S1 [file JAH3-13-e032259-s001.docx]

**Supplemental material**

Table S1. Comparison of baseline characteristics between participants included in analyses and participants without (useable) white matter change masks

|  | N=197 | N=32 | p-value |
| --- | --- | --- | --- |
| Age, mean (SD)* | 65.61 (11.10) | 67.37 (11.39) | 0.421 |
| Male sex, N (%)^$^ | 133 (67.5) | 19 (59.4) | 0.366 |
| Subcortical infarct, N (%)^$^ | 116 (58.9) | 14 (43.8) | 0.109 |
| BL WMH volume, mean (SD), ml* | 15.51 (19.23) | 11.27 (8.48) | 0.041 |

*Welch two sample t-test. ^$^Pearson’s Chi-squared test. BL: Baseline; WMH: White matter hyperintensity
